# Teaching Communication Micro-Skills to Cardiologists Managing Seriously Ill Patients in Asia: Challenges Encountered Amidst the COVID-19 Pandemic and Future Perspectives

**DOI:** 10.7759/cureus.19957

**Published:** 2021-11-28

**Authors:** Shirlyn Hui Shan Neo, Jamie X Zhou, Genevieve C Wong, Natalie K Mok, Alethea C Yee, Gillian L Phua

**Affiliations:** 1 Division of Supportive and Palliative Care, National Cancer Centre Singapore, Singapore, SGP; 2 Department of Social Work Services, National Heart Centre Singapore, Singapore, SGP

**Keywords:** clinical cardiology, end-of-life and hospice care, interprofessional education and collaboration, supportive and palliative care, patient-doctor communication

## Abstract

Background

Patients with advanced cardiac conditions value effective symptom control and empathic communication with their doctors. However, studies have shown that empathic communication with seriously ill patients does not occur adequately in cardiology. Therefore, we piloted a program for teaching communication skills in a bite-sized manner. The primary aim of the research was to understand the feasibility and acceptability of the training program and to perform a preliminary evaluation of its efficacy.

Methodology

Clinicians were recruited from the cardiology unit of a tertiary hospital in Singapore. Patients were also recruited for the audio recording of clinic consults. Recruited patients had to have a chronic cardiac condition and be deemed at risk of dying within one year. We utilized a pre-post intervention design. Prior to the educational intervention, clinicians were asked to audio record a single clinic consult at baseline. They were then asked to participate in a training program that consisted of video-annotated presentations and role-play scenarios. Subsequently, the audio recordings of their clinic consults with seriously ill patients were recorded. The audio recordings were evaluated by trainers and used for feedback with clinicians. Data on the completion rate of the training program were collected. In addition, changes in the clinicians’ self-rated communication skills and views on the acceptability and relevance of the training program were collected.

Results

Overall, five of the six clinicians (83.3%) completed all sessions in the program. One clinician only completed four out of the five sessions in the program. Clinicians deemed the program acceptable and relevant and found audio recordings to be useful for reflective learning. There was an improvement in the clinicians’ self-assessed competency. However, the planned number of audio recordings could not be completed due to the coronavirus disease 2019 pandemic.

Conclusions

The pilot training program was acceptable and relevant for the participants. However, it will require adaptation to allow it to be transferrable and scalable to all settings, especially in situations that limit prolonged face-to-face contact.

## Introduction

Patients with advanced cardiac conditions value effective symptom relief and empathic communication with their doctors [1]. However, studies of patients with serious illnesses, including cardiac disease, show that discussions of patients’ values, goals, and treatment preferences do not occur adequately [2]. A local study from Singapore showed that only 22% of advanced heart failure patients were aware that their treatments were not curative [3].

Multiple difficulties exist that prevent these medically complex conversations from happening, such as inadequate time and communication skills and personal discomfort with end-of-life conversations [1,4]. Recognizing this gap, the American Heart Association has issued a scientific statement emphasizing the importance of communication skills training for cardiologists [5].

Although a systematic review has shown that communication skills training workshops improve the communication skills of participating clinicians in oncology [6], these communication skills training programs often include face-to-face workshops using role-play and standardized patients (SPs) with a complex set of skills all taught in the same setting.

It is unknown whether the complex set of skills learned during communication skills workshops are sustained over time or translated into clinical practice. It is also currently unclear whether the content and structure of communication skills training programs developed for other disease types and settings would be culturally acceptable in the Asian cardiology care setting, where end-of-life discussions are culturally more sensitive and patients are not as ready to discuss these topics [7-9].

Study rationale

The aim of this pilot study was to pilot a communication skills training program to teach communication skills in a bite-sized, culturally sensitive, multi-modal, and blended learning manner. Our primary aim was to understand the feasibility and acceptability of the training program. Our secondary aim was to have a preliminary understanding of its efficacy.

## Materials and methods

Study setting

This study was conducted in the National Heart Centre Singapore (NHCS), a national and regional referral center for cardiovascular diseases [10].

Inclusion criteria for clinician trainees/patients

Clinicians working within the cardiology department of NHCS who were registrars (in training to become consultants) or newly certified consultants (completed fellowship exit exams within the last two years) were recruited for the study. Patients recruited for audio recordings of outpatient clinic consults had to have a chronic cardiac condition and be deemed at risk of dying within one year.

Development of training curriculum

The study team that developed the curriculum was interdisciplinary in nature and included specialty content experts in communication skills. The team consisted of a range of palliative medicine physicians of differing seniority (one associate consultant, two consultants, and one senior consultant) from the NHCS, as well as medical social workers practicing in NHCS and educators from the Duke-National University of Singapore Lien Centre for Palliative Care (LCPC). A senior consultant cardiologist acted as a specialty content expert in cardiology to ensure that the content of the training scenarios was authentic to clinical practice. The training team met twice (one hour each) prior to the start of the training program to design and structure the training curriculum.

Structure of training program curriculum (Appendices)

An audio recording of each clinician’s outpatient consults was obtained at baseline prior to starting the training program. Subsequently, the clinicians received an online link via email to watch a voice-annotated presentation for the theoretical teaching of communication micro-skills. Clinicians were given the flexibility to watch the presentation at their own time, and the only requirement was that it had to be seen before their first face-to-face training one month later.

Subsequently, clinicians had two face-to-face sessions (one hour each) at lunch break for role-play of communication scenarios, focusing on a small number of micro-skills each time. Clinicians were asked to perform one audio recording of a clinic consult between each face-to-face session. The audio recordings collected during training were used for giving feedback to the clinicians and used for comparison to the audio recordings collected prior to training commencement. The program then concluded with a short face-to-face session to consolidate learning.

Communication micro-skills taught included appropriate use of non-verbal skills, demonstration of empathy, information sharing, and eliciting of goals, values, and preferences for care. Audio recordings of consults were taken in an unscripted real-time environment. The audio recordings were reviewed by content experts from the study team and feedback on what was done well during the consult versus what could have been improved was shared with the clinicians via email.

The design of the curriculum was based on “Social Cognitive Theory” by Bandura, who proposed that new behaviors can be acquired by observing and imitating others [11], as well as on Schön’s theory of reflective practice, whereby learners would reflect both on-action and in-action [12].

Study measures and data collection

The demographic data of clinicians and patients were collected. At the end of the program, using an online survey, clinicians self-reported their views on the acceptability and relevance of the content of the program using a four-point Likert scale (strongly agree, agree, disagree, strongly disagree). They were also asked to self-assess their competency in communication skills pre- and post-training using a five-point Likert scale (extremely skilled, very skilled, skilled, somewhat skilled, not at all skilled). Responses regarding self-assessed competency were coded from 1-5, respectively, with 5 representing “a higher level of skill” and 1 representing “not at all skilled.”

Outcome assessments of the training program were based on understanding the degree to which participants found training relevant and acceptable and the degree to which participants felt that they had learned and applied new skills.

Sample size and statistical analysis

We aimed to recruit at least six clinicians as a pilot sample. This would be at least 50% of all eligible participants in the NHCS at the time of the study and would satisfy our a priori definition of feasibility. Participant demographics and study measures were summarized using descriptive statistics.

Ethics

This study was approved by the Singhealth Centralized Institutional Review Board (Approval number 2019/2570). Informed consent was obtained from all clinicians and patients.

## Results

Characteristics of participants

In total, six clinicians (mean age: 33.2 years) and 11 patient participants (mean age: 64.4 years) were recruited (Table 1).

**Table 1:** Characteristics of study participants. ^a^Mean and standard deviation for continuous variables, and frequency (N) and percentage for categorical variables.

Characteristics	Mean (standard deviation) or number (%)^a^
Clinician characteristics	
Age (years)	33.2 (1.2)
Gender (Male)	4 (66.7)
Gender (Female)	2 (33.3)
Number of years since graduating from medical school	8.3 (1.8)
Patient characteristics	
Age (years)	64.4 (4.6)
Gender (Male)	7 (63.6)
Gender (Female)	4 (36.4)
Primary cardiac diagnoses	
Ischaemic heart disease	6(54.5)
Atrial fibrillation	1 (9.1)
End-stage heart failure from non-ischemic cardiomyopathy	3 (27.3)
Chronic valvular heart disease	1 (9.1)

Impact of the training program

In total, five out of the six clinicians (83%) completed the training program, and one out of six clinicians only completed four out of five training sessions as the clinician was posted to another institution. In addition, due to the coronavirus disease 2019 (COVID-19) pandemic with its restrictions on face-to-face research, we could only complete two out of the three planned audio recording sessions and the final face-to-face teaching session had to be done virtually.

On a self-reported quantitative survey, clinicians’ responses showed that they felt that the training duration and content were acceptable and relevant and agreed that they would recommend this program to others. In addition, focusing on a few communication skills at one time rather than teaching all skills in one setting helped them to internalize learning better.

Qualitative feedback from the clinicians on audio recordings was that they felt the training was realistic with practical advice on how to improve. Moreover, it allowed them to reflect and improve on communication skills based on actual situations that they had encountered. For example, in a real-life recording where one clinician was sharing the information “It’s whether your varices have slowly bled,” the patient responded and showed difficulty understanding the use of medical jargon “When you say varices, is that the one….” This clinician was given feedback to be careful not to use medical jargon while sharing information during consultations. In another scenario, where a patient had brought up concerns over prognosis for heart failure “presently I’m not in pain, but I don’t know what will happen tomorrow, or day after tomorrow...,” another clinician responded by saying “mmm” and changed the subject. This clinician was given feedback and a sample script on how to engage the patient further to discuss prognosis and expected outcomes.

Clinicians also appreciated that the study team managed to provide a controlled and protected environment for trial and error during the role-play. This allowed for constructive learning not only from peers but also from various members of the interdisciplinary study team. Lastly, clinicians self-perceived that they had improved significantly in their communication skills post-training. Table 2 presents the clinician responses during the training program.

**Table 2:** Clinician responses regarding the training program.

Survey domains	Responses	Number of participants (%)	
Domains regarding relevance of training program (Kirkpatrick level 1)			
The program content is relevant for my practice	Strongly agree	4 (67)	-
	Agree	2 (33)	-
I will be able to apply what I was taught in my daily clinical work	Strongly agree	2 (33)	-
	Agree	4 (67)	-
Domains regarding acceptability of training program (Kirkpatrick level 1)			
Online video annotated presentation: The materials on the learning management system before the face-to-face sessions are helpful to my learning	Strongly agree	2 (33)	-
	Agree	3 (50)	-
	Disagree	1 (17)	-
Face-to-face sessions (role play): Focusing on a few communication skills at each face-to-face session rather than all skills at once helps me internalize my learning better	Strongly agree	5 (83)	-
	Agree	1 (17)	-
The face-to-face sessions allowed me to practice the communication skills in a safe environment	Strongly agree	5 (83)	-
	Agree	1 (17)	-
The facilitators provided feedback during the face-to-face session in a constructive manner	Strongly agree	6 (100)	-
Duration of programme: The time allocated for the scope of the program is feasible for busy clinicians	Strongly agree	2 (33)	-
	Agree	3 (50)	-
	Disagree	1 (17)	-
General programme: The delivery of the training program is acceptable to learners	Strongly agree	3 (50)	-
	Agree	3 (50)	-
I would recommend this program to others	Strongly agree	3 (50)	-
	Agree	3 (50)	-
Self-assessment regarding change in skills (Kirkpatrick level 2)	Pre-training median (interquartile range)	Post-training median (interquartile range)	P-value (Wilcoxon sign rank test)
Speaking to patients about their:			
Concerns and expectations	2.0 (1.0)	3.5 (1.0)	P = 0.025
Values that drive their preferences	2.0 (0.5)	3.0 (1.0)	P = 0.025
Goals of care	3.0 (1.0)	3.5 (1.0)	P = 0.046
Resuscitation status	2.0 (1.0)	3.0 (1.0)	P = 0.046
Extent of care	2.0 (1.0)	3.5 (1.0)	P = 0.025
Speaking to family about withdrawal of life-sustaining treatments for patients	2.0 (1.0)	3.0 (0.3)	P = 0.046

## Discussion

Main findings

Our pilot training program was acceptable and relevant in terms of content and was feasible for busy clinicians. There was an improvement in clinicians’ self-assessed competency of their communication skills. Audio recordings of clinic consults with feedback helped to cultivate an attitude of reflective learning. Clinicians were prompted to reflect on their clinical experiences and be more mindful of how they were communicating with their patients.

Although self-assessment of clinicians on their competencies showed improvement post-training due to the restrictions imposed by the COVID-19 pandemic, it was not possible to have prolonged face-to-face interactions, which limited our ability to formally carry out the full extent of the program.

Study strengths

To the best of our knowledge, this is the first study to evaluate the acceptability, relevance, and feasibility of teaching communication micro-skills in a cardiology unit in a tertiary hospital in Asia. We have demonstrated that it is possible to teach clinicians how to discuss culturally sensitive end-of-life topics. Although we only recruited registrars and newly certified consultants, this program can be used for teaching all cardiologists as the training content is relevant to daily clinical practice.

Study limitations

A limitation of our study was the absence of patient input regarding their communication experience with the clinicians. In addition, because clinicians self-assessed their improvement in skills, it could have introduced bias. However, an improvement in self-assessed competency implies that clinicians would be more confident and hence more likely to discuss medically complex topics with their patients in the future. Lastly, the sample size was small; however, because this was a pilot study, it did not intend to provide full insight into the effectiveness of the training program and primarily aimed to highlight the acceptability and feasibility of this program.

Suggestions for future studies

Future studies should continue to evaluate the role of reflective practice in communication training [13]. Possible adaptations of this communication skills training program could consider the videotapes of virtual consults which could allow evaluation of both verbal and non-verbal skills. In addition, the role of chatbots in communication could be further explored [14-16] as it is theoretically useful for discussions around culturally or emotionally sensitive topics, allowing ample room for trial and error on the part of the clinician, without fear of causing emotional distress on the part of the patient or family caregiver. Lastly, the use of natural language processing could be considered as it could make the process of coding conversations less resource-intensive [17].

## Conclusions

This study has shown preliminarily that teaching communication skills over time, in a bite-sized manner, utilizing different modalities of teaching, is acceptable and relevant in a busy tertiary Asian cardiology care setting and can inculcate reflective practice for continued improvement. Improving communication skills is very important as it can impact doctor-patient relationships and help patients have more realistic expectations. Future iterations of this program could consider alternate ways of conducting communication practice in view of difficulties carrying out prolonged face-to-face interactions during and after the COVID-19 pandemic.

## Appendices

**Table 3:** Summary of the training curriculum. COVID-19: coronavirus disease 2019

Timeline	Content of program	Learning objectives	Assessment methods and data collection
Session 1: Introduction session to trainees (face-to-face) regarding objectives of training program and training schedule (one hour)	Clinician trainees are introduced to the scope and structure of the program	To allow participants to understand the intention of the training program	After the introduction session concluded, a baseline demographic survey was collected. Clinicians were also asked to share their challenges with communication. One audio recording (per clinician) of a clinic consult was performed prior to the start of the training
Session 2: Clinicians are asked to watch one video-annotated presentation (VAP) (15 minutes) prior to the face-to-face sessions	Micro skills taught included (a) use of non-verbal skills (including use of appropriate body language, eye contact)	To appreciate the different types of communication micro-skills needed for communication	Trainees were sent an online link through their email for the VAP. Attendance was tracked
	Demonstrating empathy (including paraphrasing, acknowledging, and responding to emotions using the “NURSE” framework. The NURSE framework includes “Naming the emotion,” “Understanding the patient,” “Respecting the patient,” “ Supporting the patient,” “ Exploring further about the emotion”		
	Establishing perspectives (including an understanding of illness and treatment options)		
	Establishing patient’s values driving preferences and goals of care using the REMAP framework. REMAP includes “Reframe,” “Expect emotions,” “Map out the future,” “Align with patient’s goals,” “Plan treatments that match values”		
	Giving information appropriately (including giving treatment recommendations based on a patient’s values, information preferences, and goals of care)		
Session 3: First face-to-face session with role play (one hour during lunchtime)	Case scenarios: Solo discussion with the patient: Discussion with an advanced heart failure patient regarding goals of care and consideration of ventricular assist device placement	To demonstrate micro-skills learned from VAP including Non-verbal skills, demonstrating empathy, establishing perspectives, values, and goals	One audio recording (per clinician) of a clinic consult after the first face-to-face session
	Family conference: Breaking bad news to caregivers regarding a medical error	To demonstrate micro-skills learned from VAP including Empathy, giving information appropriately	
Session 4: Second face-to-face session (one hour during lunchtime)	Solo discussion with the patient: Extent of care discussion with an advanced heart failure patient who is clinically deteriorating, including consideration of future deactivation of cardiac devices	To demonstrate micro-skills learned from VAP including Non-verbal skills, demonstrating empathy, establishing perspectives, values, and goals	One audio recording (per clinician) of a clinic consult with emailed feedback after the second face-to-face session (but audio recording and patient recruitment was held off in view of the COVID-19 pandemic)
	Family conference: Breaking bad news to a family caregiver regarding the poor prognosis of an imminently dying patient in the cardiac intensive care unit including the extent of care discussion with consideration of withdrawal of life-sustaining treatment	To demonstrate empathy and give information appropriately	
Session 5: Consolidation session (planned face-to-face but was held virtually due to the COVID-19 pandemic)	Round table discussion between trainers in study team and clinician participants	To consolidate and reflect together on learning points from the program	Clinicians complete survey to feedback on acceptability, relevancy of training program and self-assess competency in communication skills
